# YUV-based SVD-VGG hybrid fusion for multimodal MRI-PET image integration

**DOI:** 10.1371/journal.pone.0340781

**Published:** 2026-01-27

**Authors:** Kandala S.S.V.V. Ramesh, S. Selva Kumar

**Affiliations:** School of Computer Science and Engineering, VIT-AP University, Beside AP Secretariat, Amaravati, Andhra Pradesh, India; Islamia University of Bahawalpur: The Islamia University of Bahawalpur Pakistan, PAKISTAN

## Abstract

Multimodal medical image fusion enhances diagnostic interpretation by integrating anatomical and functional information into a single image. This work proposes an efficient hybrid framework, termed SVD–VGG Hybrid Fusion, unifying Singular Value Decomposition (SVD) for luminance decomposition and a lightweight VGG-based feature extractor for high-frequency enhancement. Synthetic Gaussian noise ($\sigma^{2}=0.25$) is added to MRI and Poisson noise to PET images to simulate representative acquisition degradations, while the SVD and VGG-based feature paths strengthen structural detail and functional contrast. Experiments were conducted on a single public brain dataset with image pairs resized to 256×256 for fusion and 224×224 for feature extraction. Quantitative evaluation using PSNR, SSIM, CC, and perceptual LPIPS indicates that the proposed method achieves consistent structural fidelity, perceptual quality, and color preservation while maintaining sub-second runtime per case. Although evaluated only on brain MRI–PET data and under synthetic noise conditions, the results suggest that the SVD–VGG hybrid design provides a noise-aware and color-preserving fusion strategy suitable for practical multimodal image fusion workflows.

## 1 Introduction

Medical imaging is essential in clinical diagnosis, including non-invasive assessment of interior bodily systems and physiology. Various modalities, including Magnetic Resonance Imaging (MRI), Computed Tomography (CT), Positron Emission Tomography (PET), Single-Photon Emission Computed Tomography (SPECT), and Ultrasound, provide distinct diagnostic capabilities [1].

### 1.1 Background and motivation

Magnetic Resonance Imaging (MRI), Computed Tomography (CT), Positron Emission Tomography (PET), and Single Photon Emission Computed Tomography (SPECT) each offers unique diagnostic capabilities within clinical practice. MRI provides excellent soft tissue contrast and high spatial resolution making it suitable for anatomical and structural assessment. CT excels in imaging dense tissues such as bone and calcification. On the other hand, PET and SPECT provide valuable functional and metabolic insights by visualizing physiological processes such as glucose metabolism or blood flow. Despite these strengths, no single modality offers a comprehensive diagnostic view, MRI and CT lack metabolic information, whereas PET and SPECT suffer from low spatial resolution and limited anatomical context [2,3].

These limitations have catalyzed the development of Multi-Modal Medical Imaging Fusion (MMIF), which aims to integrate complementary information from multiple imaging sources into a single, more informative representation [4]. MIF plays a vital role in enhancing diagnosis by integrating complementary anatomical and functional information from modalities such as MRI and PET. Accurate preservation of PET’s metabolic color gradients is crucial since these convey functional details relevant for tumor delineation and treatment planning [5,6].

Several classical multimodal fusion techniques, particularly multiscale decomposition frameworks such as wavelet transforms [7], contourlet [8] and shearlet transforms [9], and dual-tree complex wavelets [10], have achieved promising results in enhancing detail and contrast. More recently, sparse-based hybrid models [11] and deep learning-based models [12] have been widely explored for feature-guided integration to improve texture representation and visual interpretability. While these techniques have demonstrated strong detail enhancement, multimodal MRI–PET fusion additionally requires careful preservation of metabolic color information and proper handling of modality-specific noise characteristics.

Although recent hybrid and CNN-guided fusion techniques improve detail preservation, they often fail to retain PET chrominance information since color channels are altered or fused with luminance components during synthesis [13,14]. Furthermore, modality-specific noise characteristics such as Gaussian noise in MRI and Poisson photon noise in PET are rarely addressed jointly, potentially degrading clinical interpretability under realistic acquisition conditions [6,15].

To address these challenges, this work proposes a noise-aware and color-preserving fusion framework operating in the YUV domain. The luminance channel (Y) undergoes SVD-based decomposition and lightweight VGG feature enhancement, while PET chrominance channels (U/V) are preserved intact to maintain functional color fidelity. The objective of the proposed SVD-VGG hybrid approach is to achieve computationally efficient and noise-tolerant MRI–PET fusion, improving anatomical detail and PET interpretability for clinical workflows.

## 2 Literature review

Over the years, numerous fusion strategies have emerged, spanning from traditional pixel-level averaging to advanced transform-domain techniques and modern deep learning (DL)-based frameworks [12,16]. Among classical approaches, transform-based methods such as the Discrete Wavelet Transform (DWT) [7], Contourlet Transform (CTr) [8], Nonsubsampled Contourlet Transform (NSCT) [17,18], and Shearlet-based techniques like the Nonsubsampled Shearlet Transform (NSST) [9] have demonstrated efficacy in multiscale and multidirectional decomposition, enabling effective extraction of spatial and frequency features. While DWT [19] and Dual-Tree Complex Wavelet Transform (DTCWT) [10] provide commendable frequency localization, they suffer from shift variance. In contrast, CTr and NSCT improve edge preservation but often lead to higher complexity and pseudo-Gibbs artifacts.The Gradient Pyramid (GP) [20] and Laplacian Pyramid (LP) [21] transformations enabled multi-resolution analysis but shown vulnerability to image misregistration. Fuzzy logic methodologies were developed to improve adaptability and address uncertainty. These methods utilized membership functions to depict uncertainty in pixel intensities, exhibiting effectiveness in edge preservation and contrast enhancement. Techniques encompass Intuitionistic Fuzzy Sets with Cross-Correlation (IFCC) [22], Type-2 Fuzzy Systems augmented by Teaching Learning-Based Optimization (TLBO) [23], and Undecimated Discrete Wavelet Transform (DWT) with Fuzzification [24], which have demonstrated improvements in image detail and robustness. However, these approaches were often hindered by computational complexity and optimization challenges. These traditional methods, including Principal Component Analysis (PCA) [25], are relatively effective in preserving anatomical details but frequently lack robustness against noise and perform suboptimally in handling intricate texture patterns [16]. DL-based fusion approaches, although promising, may insufficiently maintain color integrity, which is particularly crucial in functional modalities such as PET. Several advanced architectures such as ASFE-Fusion [26],MMIF-INet [27], FATFusion [28], GeSeNet [29], MATR [30], MSRPAN [31], and Zero-Learning Fusion (ZLF) [32] reflect this trade-off. ASFE-Fusion [26] adopts a dual-stream strategy combining spatial and frequency domain fusion through Cross-Attention Spatial Fusion (CASF) and Adaptive Frequency Fusion (AFF). While it achieves top-tier metrics (EN, PSNR, CC) and boosts downstream classification accuracy, it requires heavy computation (175M parameters, 112 GFLOPs), and does not address explicit chrominance preservation. FATFusion [28], based on modality-specific transformer branches and guided attention, fuses only the Y (luminance) channel of PET images while leaving chroma channels untouched, potentially discarding color cues essential for functional interpretation. GeSeNet [29] integrates semantic guidance and edge refinement for detail enhancement, but it uses multi-stage training without addressing PET color retention. MATR [30] combines adaptive convolution and multiscale transformer attention for better inter-modality feature modeling; however, it employs VGG16 backbones and only achieves moderate results on color-sensitive metrics such as LM I and MS-SSIM. MSRPAN [31], despite its efficiency and performance on SSIM and MI, only processes the Y channel of YCbCr-converted images, omitting direct chrominance fusion. ZLF [32], although extremely lightweight and training-free, extracts shallow CNN features and applies simple softmax-weighted averaging, which lacks the semantic richness and dynamic chroma handling needed for color-functional modalities like PET. MMIF-INet [27] have addressed this issue by enabling direct multichannel processing and employing hybrid loss functions to retain both color fidelity and structural detail in the fused outputs. Moreover, many contemporary models inadequately address image noise or modality-specific distortions [33]. The advent of sophisticated GPUs and deep neural networks has positioned DL-based fusion as the dominant trend. CNN-based architectures like U2Fusion [34,35] and IFCNN [36] have shown considerable effectiveness in obtaining hierarchical features and assimilating complementary input without requiring explicit rule design. Dual-discriminator GANs (DDcGAN) [37], dense residual models [38], and attention-guided fusion networks enhanced visual fidelity and feature alignment. Such limitations have prompted the exploration of more adaptable and noise-resilient techniques, paving the way for hybrid models that combine classical decomposition with data-driven deep learning for improved fusion quality.

Alongside the approaches elaborated above, various additional notable fusion techniques have significantly contributed to the advancement of MIF. Table 1 presents a thorough overview and systematic comparison of classical, fuzzy logic-based, and deep learning-based fusion approaches. This table outlines the key characteristics, benefits, and limitations of many approaches frequently referenced in the literature, serving as a foundation for evaluating the efficacy and relevance of the proposed model.

**Table 1:** Summary of representative medical image fusion methods.

Method	Core Strategy	Strengths	Limitations
UDWT + Fuzzification [24]	Wavelet-domain fusion with fuzzy rule-based region selection	Improves contrast and manages uncertainty in low-contrast regions	High computational cost; sensitive to fuzzy parameter selection
EN + GCF [39]	Entropy-driven filtering with gradient-based fusion	Enhances structural details and local contrast	Algorithmically complex; limited robustness under noise
FCM + DCT-FT [40]	Hybrid clustering and frequency-domain fusion	Preserves structural and spectral information	Computationally intensive; parameter-sensitive
FCN-based Fusion [41]	End-to-end CNN-based image fusion	Automatically learns fusion rules; avoids hand-crafted features	Requires large training datasets; high training cost
Multi-Scale CNN [11]	Multi-resolution CNN feature extraction and fusion	Captures both local and global features	High computational and training overhead
RFN [38]	Residual feature learning for fusion	Emphasizes salient inter-modality differences	Data-hungry; risk of overfitting on limited datasets
NSST + Frei-Chen Masks [42]	Shearlet-domain fusion with edge-focused masks	Strong texture and edge preservation	Complex edge detection; manual mask tuning required

While U2Fusion [35] adaptively fuses multiple modalities without ground truth, it processes only the Y channel in YCbCr and averages chrominance, losing spatial color gradients. GeSeNet [29] achieves ultrafast, artifact-reduced fusion via a semantic-guided mask ensemble but relies on two-stage training with multiple hyper parameters. FATFusion [28] captures functional and anatomical features through dual transformer branches in YCbCr yet demands high GPU memory, extensive training data, and still fuses only luminance. Deep learning has emerged as a leading strategy for medical image fusion by leveraging pretrained feature extraction networks such as VGG and ResNet to improve perceptual structure transfer. Some approaches combine deep features with optimization-driven refinements for contrast enhancement and detail balancing. For instance, MIF-BTF-MRN performs a bilateral texture decomposition and utilizes a transfer-learned ResNet-101 backbone to guide detail preservation, while the Crayfish/Coati Optimization Algorithm (COA) adapts base-layer fusion weighting [43]. Similarly, the EOA-based Adaptive Three-Component Image Decomposition (ATCID) method computes Multi-Feature Local Energy (MFLE) for detailed layers and optimizes low-frequency fusion using the Equilibrium Optimization Algorithm (EOA) to maintain structural consistency [44]. Although these models improve fine-texture representation, their optimization-intensive pipelines may result in increased runtime and they do not explicitly address different noise characteristics present in MRI–PET pairs. The TL-VGG19 fusion method also employs a YUV-based strategy for multimodal fusion, where the color image is converted to the YUV space and only the luminance channel (Y) is fused using transformer-based low-frequency prediction and VGG19-guided high-frequency enhancement [45]. The original chrominance components (U/V) are retained and reattached to produce a color-preserved fused output. While this solution preserves metabolic color information, the authors note that the patch-based iterative modules increase computational burden, particularly at larger window sizes. In contrast, our SVD-VGG hybrid method adopts a closed-form SVD decomposition for luminance enhancement and a lightweight VGG19-driven gating mechanism that avoids nuclear-norm or metaheuristic optimization. Additionally, we explicitly incorporate Gaussian noise for MRI and Poisson noise for PET in the fusion process, improving visual stability under realistic acquisition conditions while ensuring PET chrominance fidelity.

**Key Contributions.** The key contributions of this work are summarized as follows:

- A color-preserving fusion strategy operating in the YUV space, where original PET chrominance (U/V) channels are retained completely to maintain metabolic information.
- A hybrid luminance enhancement design combining closed-form SVD decomposition of low-frequency structure with a lightweight VGG19-guided gating mechanism to selectively reinforce high-frequency detail.
- A noise-aware preprocessing stage that handles Gaussian noise in MRI and Poisson photon noise in PET to achieve improved visual stability under realistic acquisition conditions.
- A computationally efficient pipeline that avoids patch-wise nuclear-norm optimization and transformer blocks, enabling sub-second runtime per fused case on standard hardware.
- A comprehensive evaluation on brain MRI–PET data using pixel-, structure-, and perception-based metrics confirming improved structural detail and functional interpretability.

The remainder of this paper is organized as follows: Sect 3 presents the proposed methodology, including preprocessing, decomposition, feature extraction, and fusion strategies. Sect 4 reports the experimental results and provides a comparative evaluation using established performance metrics and statistical tests. Sect 5 discusses the limitations of the current work and outlines future directions. Finally, Sect 6 concludes the study by summarizing the key findings and overall contributions.

## 3 Methodology

The proposed SVD-VGG fusion framework is designed to preserve the metabolic chrominance of PET while enhancing the anatomical luminance detail from MRI in the YUV domain. The luminance channel (Y) is strengthened using SVD–based decomposition and lightweight VGG19-driven high-frequency feature modulation, whereas the original PET chrominance channels (U/V) are retained to maintain functional color fidelity.

**Fig 1:**
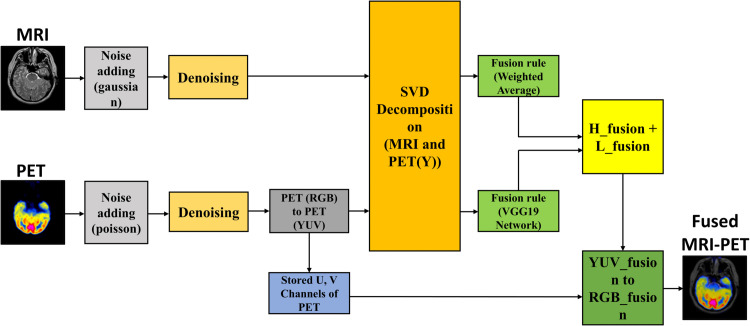
Overall architecture of the proposed method.

This section establishes the methodology of the proposed MIF approach, which amalgamates SVD and a trained deep learning-based feature extractor (VGG19). The procedure encompasses noise simulation, hybrid denoising, image decomposition, deep feature extraction, fusion approach, and evaluation. Fig 1 depicts the comprehensive process of the proposed SVD-VGG MIF model. It offers an overview of the pre-processing, decomposition, feature extraction, and reconstruction phases involved in our hybrid workflow.

### 3.1 Preprocessing and noise modeling

In real-world clinical settings, medical images inherently suffer from noise introduced during acquisition and transmission. To evaluate the effectiveness and reliability of the proposed fusion and denoising pipeline under diverse noise conditions, this study employs a controlled synthetic noise simulation approach. Rather than relying on variable real clinical acquisitions with unknown and uncontrollable noise characteristics, synthetic noise is systematically added to clean baseline images from standard public datasets. This controlled-noise methodology is well-established in medical imaging research [46] as it enables: (1) ground-truth reference availability for quantitative evaluation, (2) systematic testing across defined and reproducible noise levels, and (3) direct comparison with state-of-the-art methods using identical noisy inputs, eliminating confounding factors from variable real acquisitions.

The source MRI and PET images used in this study are noise-free reference datasets obtained from the Harvard Medical Image Fusion Dataset repository. To simulate realistic clinical noise degradation, synthetic noise is externally applied post-acquisition. Specifically, Gaussian noise [47], which simulates scanner-induced statistical fluctuations commonly observed in MRI images, is synthetically generated and added to the clean MRI source. Meanwhile, PET images are subjected to Poisson noise (also known as photon noise) [47], which is synthetically generated to simulate the stochastic nature of radioactive decay and photon detection.

**Fig 2:**
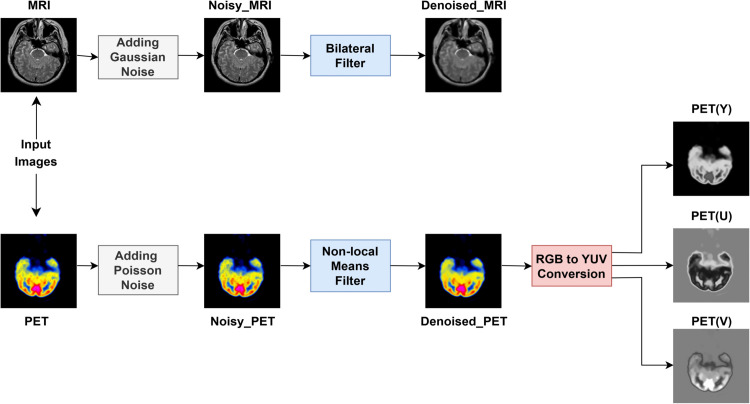
Image pre-processing.

A hybrid denoising method is utilized to alleviate these distortions and maintain image integrity prior to fusion. Fig 2 visually depicts the entire workflow of these preprocessing activities. Bilateral filtering is employed in MRI images to enhance smoothness while preserving edges [48]. PET scans are subjected to a two-phase denoising procedure that combines Non-Local Means (NLM) filtering, which utilizes patch similarity, and guided filtering, which improves details through a guiding picture. This guarantees that the inputs to the fusion network are pristine, edge-preserving, and clinically dependable. In MMIF, particularly when amalgamating color-dense PET images with grayscale MRI, it is imperative to maintain both structural and chromatic information. To achieve this, PET images are converted from RGB to YUV color space, enabling the distinction of luminance (Y) and chrominance (U, V) components. This segregation facilitates precise processing, such as structural fusion in the Y channel, while preserving color accuracy via the U and V channels. The YUV color space enables separate handling of luminance (Y) and chrominance (U, V), aligning with our design where structural fusion is best applied on intensity (Y), while U and V preserve PET color fidelity. This separation facilitates modality-specific processing and reduces redundancy compared to RGB fusion, making it particularly well-suited for multimodal fusion involving color-sensitive modalities like PET.

**Fig 3:**
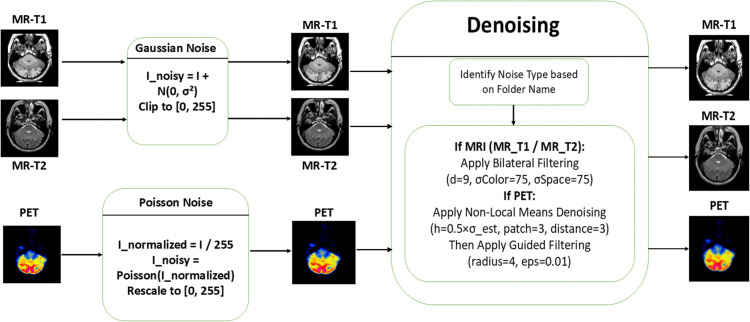
Noising and denoising module.

Two kinds of synthetic noise were added to the input photos before fusion to improve the reliability of the fusion pipeline and evaluate performance under real-world noise situations. Fig 3 offers a visual description of the noise modelling and denoising pathway used on PET and MRI data.

**Gaussian noise for MRI (MR_T1 and MR_T2):**

$$I_{g}=I+𝒩(0,\sigma^{2})$$ (1)

Where *I* is the original image and $𝒩(0,\sigma^{2})$ is Gaussian noise with zero mean and variance $\sigma^{2}=0.25$. The variance parameter $\sigma^{2}$ is a fundamental statistical measure of noise intensity, mathematically defined as the square of the standard deviation: $\text{var}(X)=\sigma^{2}$. Therefore, directly increasing variance proportionally increases the level of noise degradation in images [46]. In accordance with standard denoising and noise-modeling practice, the impact of Gaussian noise on MRI images depends directly on its variance $\sigma^{2}$, where larger values induce proportionally stronger degradation of anatomical edges and fine-scale structure [46,47]. This behavior is extensively documented in denoising literature, where multiple noise levels, σ∈{15,25,50,75} are routinely evaluated to study PSNR/SSIM degradation and denoising effectiveness [49]. Following this established methodology, we also examined several variance values $\sigma^{2}\in \{0.01,0.04,0.09,0.16,0.25\}$ to characterize noise sensitivity in our MRI–PET fusion pipeline.

**Poisson Noise (Photon noise) for PET:**

$$I_{p}=\text{Poisson}(I)$$ (2)

This reflects the statistical variation in photon emission and detection in PET imaging system [47].

#### 3.1.1 Noise-variance sensitivity rationale.

In accordance with standard denoising and noise-modeling practice, the impact of Gaussian noise on MRI images depends directly on its variance $\sigma^{2}$, where larger values induce proportionally stronger degradation of anatomical edges and fine-scale structure. This behavior is extensively documented in denoising literature, where multiple noise levels (e.g., σ∈{30,50,75}) are routinely evaluated to study PSNR/SSIM degradation and denoising effectiveness [49]. Following this established methodology, we also examined several variance values $\sigma^{2}\in \{0.01,0.04,0.09,0.16,0.25\}$ to characterize noise sensitivity in our MRI–PET fusion pipeline. This analysis verifies that $\sigma^{2}=0.25$ serves as a meaningful upper-noise condition representative of challenging but clinically plausible MRI acquisition scenarios, thereby justifying its selection for the main experiments.

**Denoising:** To store visual quality while preserving fine details and edges, a hybrid denoising strategy was applied:

**MRI – Bilateral Filtering [48]:** A non-linear, edge-preserving filter:

$$I_{d}(x)=\frac{1}{W(x)}\sum_{i\in \Omega }G_{s}(\|x-i\|)\cdot G_{r}(|I(x)-I(i)|)\cdot I(i)$$ (3)

where:

*I*_*d*_(*x*): denoised intensity at pixel *x*,

*G*_*s*_, *G*_*r*_ are spatial and range Gaussian kernels,

Ω is the neighbourhood window,

*W*(*x*) is the normalization factor,

*I*(*x*) is the intensity of neighbouring pixel *x*.

This non-linear filter smooths the image while preserving edges using spatial and range Gaussian kernels [50,51].

**PET – NLM + Guided Filtering:**

**NLM Filtering [50]:**

$$\hat{I}(x)=\sum_{i\in \Omega }w(x,i)\cdot I(i)$$ (4)

where:

$\hat{I}(x)$: denoised intensity at pixel *x*,

Ω: search window around *x*,

*I*(*i*): intensity value of neighbouring pixel *i*,

*w*(*x*, *i*): similarity-based weight between pixels *x* and *i*, defined as:

$$w(x,i)=\frac{1}{Z(x)}\exp\left(-\frac{\|P_{x}-P_{i}{\|}_{2}^{2}}{h^{2}}\right)$$ (5)

where:

*P*_*x*_, *P*_*i*_ are image patches around pixels *x* and *i*,

*h* is the filtering strength,

*Z*(*x*) is a normalization term.

This method uses patch similarity to compute weighted averaging for denoising [50].

**Guided Filtering [52]**

$$q_{i}=a_{k}I_{i}+b_{k},\;\forall i\in w_{k}$$ (10)

where:

*q*_*i*_: filtered image intensity at pixel *i*,

*I*_*i*_: guidance image intensity at *i*,

*a*_*k*_, *b*_*k*_: local linear coefficients in window *w*_*k*_.

These coefficients are computed by minimizing:

$$R(a_{k},b_{k})=\sum_{i\in w_{k}}\left((a_{k}I_{i}+b_{k}-p_{i}{)}^{2}+\epsilon a_{k}^{2}\right)$$ (6)

where:

*p*_*i*_: input noisy image,

*ε*: regularization parameter.

Guided filtering enforces structural preservation using the input image as a reference guide [52].

### 3.2 Fusion in YUV color space

Explicitly convert RGB PET images into YUV color space to separate structural (Y) and chromatic (UV) information clearly:

**Explicit RGB to YUV.** The RGB to YUV color space conversion can be represented as:

$$\left[\begin{array}{c} Y\\ U\\ V \end{array}]=[\begin{array}{ccc} 0.299 & 0.587 & 0.114\\ -0.147 & -0.289 & 0.436\\ 0.615 & -0.1515 & -0.100 \end{array}][\begin{array}{c} R\\ G\\ B \end{array}\right]$$ (7)

**Explicit YUV to RGB (Inverse clearly).** The YUV to RGB conversion is given by:

$$\left[\begin{array}{c} R\\ G\\ B \end{array}]=[\begin{array}{ccc} 1 & 0 & 1.140\\ 1 & -0.395 & -0.581\\ 1 & 2.032 & 0 \end{array}][\begin{array}{c} Y\\ U\\ V \end{array}\right]$$ (8)

The pre-processing step therefore not only converts the images into appropriate color space representation (RGB to YUV) but also enhances their quality through noise correction, setting a solid foundation for decomposition and feature extraction.

### 3.3 Feature extraction using trained VGG19

In this work, domain-specific feature are extracted from pre-processed MRI and PET images using a customized VGG19 network. Only the convolutional backbone VGG19 is kept and refined after the usual fully connected classification layers are eliminated. In order to maintain spatial dimensions, all of the convolutional layers in VGG19 [45] employ 3×3 kernels with padding and stride of 1. These kernels, often referred to as learnable filters, are initially trained using weights that were learned on ImageNet and subsequently adjusted through task-specific training. The number of kernels increases with network depth, from 64 in the first block to 512 in the following layers. This allows the network to collect increasingly complicated and abstract data that is pertinent to multimodal medical imaging. MaxPooling operations with 2 × 2 kernels and stride of 2 are applied after each set of convolutional layers. These learnable actions increase the receptive field and boost computing efficiency by halving spatial resolution while keeping the most prominent activations.

Each convolutional kernel generates a feature map that highlights regional patterns such as structural contours, edges, and textures. A more spatially rich and hierarchical representation of the input is obtained by consolidating the feature maps along the channel axis. VGG19 can effectively extract modality-specific and spatially aligned features from MRI and PET images because to this architecture. The choice of VGG19 as the backbone is motivated by its ability to provide dense low-level and mid-level texture representations that transfer well to multimodal brain MRI-PET data. Its convolutional blocks, initially trained on ImageNet, emphasize edges, contours, and local patterns, which are critical for preserving anatomical structures and metabolic gradients in fusion. Moreover, when used purely as a convolutional feature extractor without the fully connected classification head, VGG19 yields compact 512-channel feature maps at a fixed 224×224 input resolution, resulting in a relatively low-dimensional feature representation that is computationally efficient for the subsequent SVD-based fusion stage. In contrast, lighter or residual backbones such as ResNet-variants [53] typically produce higher-dimensional, more semantically oriented feature embeddings that are better suited for classification tasks than for pixel-level medical image fusion, where fine anatomical detail and texture fidelity are paramount. By applying a set of data augmentation techniques during training, the feature extractor is further adapted for the medical imaging domain. Resized to 224 × 224 pixels, each image undergoes Gaussian blur, affine transformations, color jittering (hue, brightness, contrast, and saturation), and random horizontal flipping. In terms of mean and standard deviation, pixel intensities are normalized to zero mean and unit variance. L1 feature consistency loss, which promotes stability in the deep features taken from the original image and its augmented counterpart, is used to establish the training target. The network is trained over 10 epochs using the Adam optimizer, which has a learning rate of 1 × 10^−5^. The VGG19 feature extractor is trained on the 269 paired brain MRI–PET images from the Harvard Medical Image Fusion Dataset described in Sect 4, whereas the 94 T1-PET and 94 T2-PET pairs from the Harvard Medical School archive are strictly held out for testing and are never used during VGG19 training or hyperparameter selection. Although the medical training set is modest in size, overfitting is mitigated by three factors: (i) only the convolutional backbone of VGG19 is retained and initialized with ImageNet-pretrained weights, which substantially reduces the effective parameter search space compared with training from scratch; (ii) extensive domain-specific data augmentation (random horizontal flip, random rotation within $\pm 15^{\circ }$, ColorJitter in brightness, contrast, hue, and saturation, and Gaussian blur) increases the diversity of the training samples and improves robustness to acquisition variability; and (iii) conservative optimization using a small learning rate (1 × 10^−5^), StepLR decay, and gradient clipping stabilizes training such that 10 epochs are sufficient to reach a practical performance plateau without observable overfitting in our experiments. The learning rate is gradually reduced throughout training using a StepLR scheduler with a step size of 3 and a decay factor of 0.7. Gradient clipping is used with a maximum norm of 1.0 to prevent gradient explosion. The extensive training architecture enables the network to learn robust and modality-specific semantic properties that are essential for effective MIF. While the L1 consistency loss does not directly encode fusion-task supervision, this design is deliberate for three reasons. First, domain-adaptive training on paired MRI-PET data with medical-specific augmentations (RandomRotation, ColorJitter, GaussianBlur) ensures that the VGG19 extractor learns modality-specific representations adapted to clinical imaging variations rather than generic patterns. Second, the hybrid architecture itself provides implicit fusion guidance: SVD handles structural decomposition with explicit rules, YUV space isolation protects PET chrominance, and the scalar gating mechanism constrains feature outputs to single-weight modulation. These architectural constraints channel self-consistency learning toward fusion-relevant extraction. Third, empirical validation through the ablation study shows that VGG-only fusion achieves an SSIM of 0.8777 for T1-PET, which is approximately three times higher than the SVD-only configuration (0.2943), confirming that self-consistency-trained features effectively facilitate fusion despite the absence of explicit supervision.

**Fig 4:**
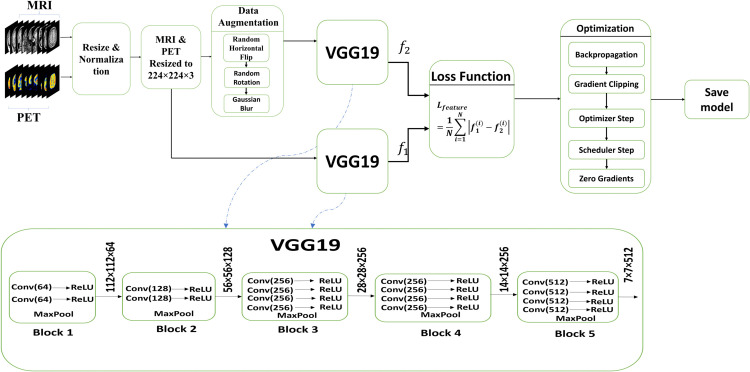
VGG19 network training.

A detailed overview of the kernel configuration is presented in Table 2, while the spatial dimensions of feature maps across the network layers are visually illustrated in Fig 4, offering clear insight into the depth-wise and spatial progression of the learned representations. To improve feature generalization across modality variations, the VGG19 [45] model is trained using domain-specific augmentations. The training process is structured in the algorithm 1

**Table 2:** Detailed kernel and feature map configuration in VGG19.

Block	Layer	Kernel Size	Input Channels	No. of Kernels	Output Feature Maps
1	Conv-1	3×3	3	64	64
	Conv-2	3×3	64	64	64
2	Conv-3	3×3	64	128	128
	Conv-4	3×3	128	128	128
3	Conv-5	3×3	128	256	256
	Conv-6	3×3	256	256	256
	Conv-7	3×3	256	256	256
	Conv-8	3×3	256	256	256
4	Conv-9	3×3	256	512	512
	Conv-10	3×3	512	512	512
	Conv-11	3×3	512	512	512
	Conv-12	3×3	512	512	512
5	Conv-13	3×3	512	512	512
	Conv-14	3×3	512	512	512
	Conv-15	3×3	512	512	512
	Conv-16	3×3	512	512	512

This training ensures that the VGG19 feature extractor captures modality-specific semantic representations robust to noise and contrast variations. The next stage utilizes these learned features for guiding high-frequency fusion.

The image pixel values are normalized to the range [–1,1] using:

$$I_{\text{norm}}=\frac{I-0.5}{0.5}$$ (9)

**Data Augmentation:** The training images undergo the following transformations:

- Resizing to 224×224
- Random horizontal flip (probability = 0.5)
- Random rotation within $\pm 15^{\circ }$
- Color jitter (brightness, contrast, hue, saturation)
- Gaussian blur with σ∈[0.1,2.0]

where ϕ(·) denotes the feature extractor (VGG19), and $I_{\text{org}}$, $I_{\text{aug}}$ are the original and augmented images, respectively.

**Optimizer:** Adam optimizer is used with a learning rate of $1\times 10^{-5}$.

**Learning Rate Scheduler:** A StepLR scheduler with a step size of 3 and decay factor of 0.7 is applied.

**Gradient Clipping:** To prevent exploding gradients, the maximum gradient norm is clipped to 1.0.

**Training Duration:** The model is trained for 10 epochs.

**Algorithm 1. Training of VGG19 feature extractor.**



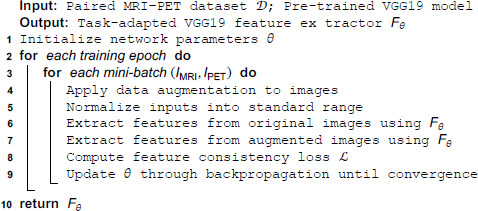



#### 3.3.1 Feature consistency loss function.

To enforce semantic stability across augmentations, an L1-based feature consistency loss is employed:

$${ℒ}_{f}={\left\|\phi (I_{\text{orig}})-\phi (I_{\text{aug}})\right\|}_{1}$$ (10)

where ϕ(·) denotes the VGG19-based feature extractor, and $I_{\text{orig}},I_{\text{aug}}$ are the original and augmented inputs respectively.

### 3.4 Image decomposition using SVD

**Fig 5:**
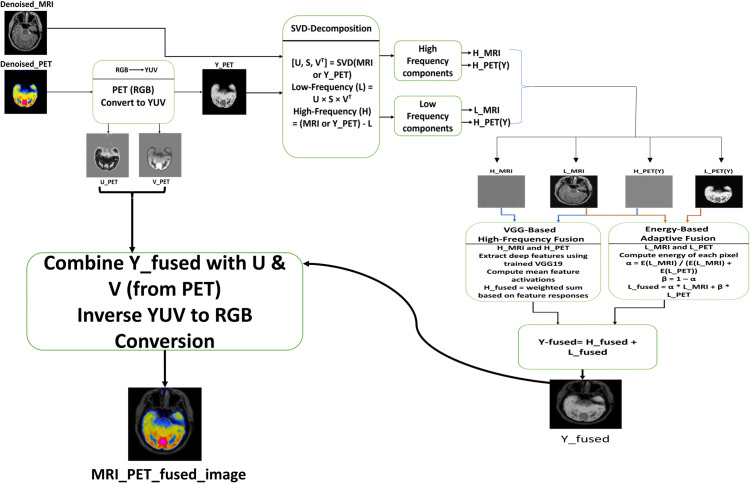
Method architecture.

Fig 5 shows the whole fusion pipeline comprising decomposition, feature-based fusion, and reconstruction. SVD decomposes both MRI and PET images into low-frequency (LF) and high-frequency (HF) components to isolate structural and detailed content. SVD is applied to the Y(luminance) channel of the PET image, extracted after converting RGB to YUV. For MRI, which is already grayscale, SVD is directly applied to the intensity values.

**SVD:**

$$I=U\Sigma V^{T}$$ (11)

where:

*I*: input grayscale image,

*U*: orthonormal matrix (left singular vectors),

Σ: diagonal matrix of singular values,

*V^T^*: orthonormal matrix (right singular vectors).

**Component Separation:**

$$L=U\Sigma_{k}V^{T}$$ (12)

H=I−L (13)

where:

$\Sigma_{k}$: retains only the top *k* singular values,

*H*: captures the discarded details. To determine whether truncated SVD provides any benefit, we evaluated several truncation ranks k∈{10,20,50,100,150} and compared them against the full decomposition. The results (Table 7) show that small ranks (e.g., *k* = 10–20) lead to substantial degradation in fusion quality, particularly in SSIM, due to loss of anatomical structure. Increasing the rank to *k* = 50–100 improves performance markedly; however, for k≥150, the performance becomes nearly identical to that of full SVD, with differences below 0.0002 in SSIM and below 0.02 dB in PSNR. This indicates that once the majority of singular energy is preserved, truncation offers negligible additional benefit. Given the minimal performance gain from tuning *k* beyond 100 and the added hyperparameter complexity, the full SVD is adopted in the proposed method for maximum stability, reproducibility, and elimination of rank-selection sensitivity. Importantly, the computational cost between *k* = 150 and full SVD is nearly identical, since both require reconstruction using a large portion of the singular spectrum. Therefore, the full decomposition is used throughout the pipeline.

### 3.5 Fusion strategy

After denoising and decomposition, a two-stage fusion approach is applied.

**LF Fusion (L-components).** LF components $L_{\text{MRI}}$ and $L_{\text{PET}}$ contain structural information. An adaptive weighting strategy based on energy distribution is applied:

$$E_{L}=\sum_{i,j}L(i,j{)}^{2}$$ (14)

$$\alpha =\frac{E_{L_{\text{MRI}}}}{E_{L_{\text{MRI}}}+E_{L_{\text{PET}}}},\;\beta =1-\alpha$$ (15)

The fused LF component is given by:

$$L_{f}=\alpha \cdot L_{\text{MRI}}+\beta \cdot L_{\text{PET}}$$ (16)

where:

*E*_*L*_ = total energy of the LF component,

*L*(*i*, *j*) = intensity at pixel (*i*, *j*),

*α* and *β* = adaptive fusion weights,

$E_{L_{\text{MRI}}}$, $E_{L_{\text{PET}}}$ = energy values of MRI and PET components, respectively.

**HF Fusion (HF-components).** HF components $H_{\text{MRI}}$ and $H_{\text{PET}}$ contain edge and texture details. Instead of direct averaging, deep features from the trained VGG19 model are used to weight fusion:

$$H_{f}=\left(\frac{\mu_{H_{\text{MRI}}}+\mu_{H_{\text{PET}}}}{2}\right)\cdot H_{\text{PET}}$$ (17)

where:

*H*_*f*_ = fused HF component,

$\mu_{H_{\text{MRI}}}$, $\mu_{H_{\text{PET}}}$ = mean feature activations extracted from VGG19.

This approach leverages deep learning to enhance fine details in the fused image.

**Image Reconstruction.** After fusion, the final fused intensity image is reconstructed as:

$$Y_{f}=L_{f}+H_{f}$$ (18)

where:

*Y*_*f*_ = final fused intensity image.

This is then recombined with the *U* and *V* channels (from PET YUV conversion) and transformed back to RGB using inverse YUV conversion, ensuring realistic color representation. The overall fusion pipeline is modularized into clearly defined steps encompassing preprocessing, decomposition, adaptive fusion, and reconstruction. The structured pseudocode is represented in algorithm 2

**Algorithm 2. SVD-VGG fusion of MRI and PET images.**

  **Input:** Paired MRI and PET images; trained feature extractor $F_{\theta }$

  **Output:** Fused RGB image

**1** Convert PET image to YUV and extract (*Y*, *U*, *V*)

**2** Convert MRI to grayscale intensity $Y_{MRI}$

**3** Decompose luminance channels using SVD:

**4**   $\left(L_{MRI},H_{MRI}\right)$, $\left(L_{PET},H_{PET}\right)$

**5** Compute adaptive weights using LF energy

**6** Fuse LF components: $L_{f}\leftarrow \alpha L_{MRI}+(1-\alpha )L_{PET}$

**7** Extract deep features from HF components via $F_{\theta }$

**8** Generate fused HF based on feature modulation

**9** Reconstruct fused luminance $Y_{f}\leftarrow L_{f}+H_{f}$

**10** Combine *Y*_*f*_ with original (*U*, *V*) and convert back to RGB

**11 return** *Fused image*

*Complexity (code-level):* pixelwise/SVD steps on the luminance image are 𝒪(N) at the working resolution; the VGG stage runs at a fixed 224×224 input.

This modular design ensures clarity in execution and supports easy extension or replacement of individual stages. The fusion process retains structural, color, and contextual details effectively through this hybrid pipeline.

### 3.6 Performance metrics

The following standard measures are employed to explicitly evaluate and logically compare the quality and performance of image fusion:

**Entropy (EN).** EN measures the informational richness of the fused image. A higher EN value implies more detail and variation in pixel intensities [54].

$$\text{EN}=-\sum_{i=0}^{L-1}P_{r}(i)\log_{2}P_{r}(i)$$ (19)

where:

*L*: total number of gray levels (typically 256 for 8-bit images),

*P*_*r*_(*i*): probability of occurrence of gray level *i*.

**Standard Deviation (SD).** SD evaluates the variation in intensity (contrast) within the image. A higher SD signifies higher contrast [54].

$$\text{SD}=\sqrt{\frac{1}{MN}\sum_{i=1}^{M}\sum_{j=1}^{N}{\left(f(i,j)-\mu \right)}^{2}}$$ (20)

where:

*M*, *N*: number of rows and columns of the image,

*f*(*i*, *j*): pixel intensity at position (*i*, *j*),

*μ*: mean intensity of the image, given by:

$$\mu =\frac{1}{MN}\sum_{i=1}^{M}\sum_{j=1}^{N}f(i,j)$$ (21)

**Average Gradient (AG).** AG assesses the sharpness and edge information in the image. A higher AG indicates clearer edges [54].

$$\text{AG}=\frac{1}{\left(M-1)(N-1\right)}\sum_{s=1}^{M-1}\sum_{t=1}^{N-1}\sqrt{\frac{\Delta_{x}(s,t{)}^{2}+\Delta_{y}(s,t{)}^{2}}{2}}$$ (22)

where:

$\Delta_{x}(s,t)=f(s+1,t)-f(s,t)$: horizontal gradient,

$\Delta_{y}(s,t)=f(s,t+1)-f(s,t)$: vertical gradient,

*f*(*s*, *t*): pixel value at local (*s*, *t*),

*M*, *N*: number of rows and columns of the image.

**Mean Square Error (MSE).** The MSE quantifies the average squared deviation of the fused image from the Ground Truth (GT) image. Lower MSE indicates better similarity [54].

$$\text{MSE}=\frac{1}{MN}\sum_{i=1}^{MN}(R_{i}-F_{i}{)}^{2}$$ (23)

where:

*R*_*i*_: pixel value of the GT (reference) image at index *i*,

*F*_*i*_: pixel value of the fused image at index *i*,

*MN*: total number of pixels.

**Peak Signal-to-Noise Ratio (PSNR).** PSNR assesses the reconstruction quality of the fused image in comparison to the GT. An increased PSNR signifies superior quality [54].

$$\text{PSNR}=10\log_{10}\left(\frac{{\text{MAX}}^{2}}{\text{MSE}}\right)$$ (24)

where:

MAX: maximum possible pixel intensity (255 for 8-bit images),

MSE: Mean Square Error.

**Structural Similarity Index (SSIM).** SSIM measures perceived image quality analyzing the structure, contrast, and brightness of the fused image with the GT image [54].

$$\text{SSIM}=\frac{\left(2\mu_{F}\mu_{I}+C_{1})(2\sigma_{FI}+C_{2}\right)}{\left(\mu_{F}^{2}+\mu_{I}^{2}+C_{1})(\sigma_{F}^{2}+\sigma_{I}^{2}+C_{2}\right)}$$ (25)

where:

$\mu_{I}$, $\mu_{F}$: mean pixel intensities of images *I* (original) and *F* (fused),

$\sigma_{I}^{2}$, $\sigma_{F}^{2}$: variances of *I* and *F*,

$\sigma_{FI}$: covariance between *I* and *F*,

*C*_1_, *C*_2_: stabilizing constants.

**Correlation Coefficient (CC).** CC measures the linear correlation between the fused and GT images. Values approaching 1 signify greater similarity [54].

$$\text{CC}=\frac{\sum_{i=1}^{MN}(R_{i}-\mu_{R})(F_{i}-\mu_{F})}{MN\cdot \sigma_{R}\sigma_{F}}$$ (26)

where:

*R*_*i*_, *F*_*i*_: pixel intensities of GT and fused images,

$\mu_{R}$, $\mu_{F}$: mean intensities of *R* and *F*,

$\sigma_{R}$, $\sigma_{F}$: standard deviations of *R* and *F*,

*MN*: total number of pixels.

**Learned Perceptual Image Patch Similarity (LPIPS).** LPIPS quantifies perceptual similarity [33] by comparing deep features from a pretrained convolutional neural network (e.g., VGG, AlexNet). Unlike pixel-wise metrics such as PSNR or SSIM, LPIPS better reflects human visual perception, making it a valuable metric for assessing image fusion quality in clinical settings [55,56].

**LPIPS is defined as:**

$$\text{LPIPS}(I_{1},I_{2})=\sum_{l}\frac{1}{H_{l}W_{l}}\sum_{h,w}{\left\|w_{l}⊙\left(F_{l}^{\left(1\right)}(h,w)-F_{l}^{\left(2\right)}(h,w)\right)\right\|}_{2}^{2}$$ (27)

where $F_{l}^{\left(i\right)}$ denotes the feature map of image *i* at the *l*-th layer of a pretrained network, *H*_*l*_, *W*_*l*_ are spatial dimensions, and *w*_*l*_ are learned channel-wise weights. The operator ⊙ represents element-wise multiplication.

**Multi-Reference Evaluation:**

$${\text{LPIPS}}_{\text{Ref}}=\text{LPIPS}(I_{\text{fused}},I_{\text{reference}})$$ (28)

$${\text{LPIPS}}_{\text{PET}}=\text{LPIPS}(I_{\text{fused}},I_{\text{PET}})$$ (29)

$${\text{LPIPS}}_{\text{MRI}}=\text{LPIPS}(I_{\text{fused}},I_{\text{MRI}})$$ (30)

Lower LPIPS values indicate higher perceptual similarity. In our study, all three comparisons are reported to evaluate perceptual fidelity of the fused images.

### 3.7 Implementation environment and hardware specifications

Training was conducted on Google Colaboratory (Python 3.8, PyTorch 1.10) using an NVIDIA Tesla T4 GPU (16 GB GDDR6, 2,560 CUDA cores, 320 Tensor Cores), Intel Xeon CPU (2.3 GHz, 2 cores), and 12 GB RAM. The model was trained for 10 epochs with an initial learning rate of 1 × 10^−5^, a StepLR scheduler (step size = 3, γ=0.7), and gradient clipping (max-norm = 1.0).

Inference was performed on a local HP Laptop 15s-ey2xxx (Windows 11 Home, AMD Ryzen 3 5300U @ 2.60 GHz, 4 cores, 8 GB DDR4 RAM) using Python 3.x with OpenCV 4.x, NumPy 1.x, Pandas 1.x, and LPIPS 0.1. The average end-to-end inference time for a single 224×224 MRI–PET pair was approximately 0.56 seconds, demonstrating real-time capability on standard CPU hardware.

### 3.8 Computational complexity analysis

The computational complexity of the proposed SVD–VGG fusion pipeline is analyzed by decomposing it into three main stages: luminance decomposition, deep feature extraction, and fusion–reconstruction.

**Decomposition Phase:** The Singular Value Decomposition (SVD) of an image matrix of size *M* × *M* has a theoretical time complexity of *O*(*M*^3^). In the proposed method, SVD is applied to fixed-resolution medical image slices of size 256 × 256, which are commonly used in slice-based multimodal medical image fusion. As a result, the SVD stage incurs a *fixed per-image computational cost under the working resolution used in this study*. Although this complexity is superlinear with respect to the number of pixels *N* = *M*^2^, it remains practical and manageable for 2D fusion scenarios and avoids the need for iterative optimization, nuclear-norm minimization, or patch-wise decomposition.

**Feature Extraction Phase:** The VGG19-based feature extractor operates on inputs resized to a fixed spatial resolution of 224×224, independent of the original image dimensions. During inference, only the convolutional backbone of VGG19 is used, and no backpropagation or iterative refinement is performed. Consequently, the feature extraction stage incurs a constant inference cost per image pair, denoted as $O(C_{\text{VGG}})$, where $C_{\text{VGG}}$ represents the fixed number of operations in a forward pass of the truncated VGG network at the specified resolution.

**Fusion and Reconstruction Phase:** The computation of adaptive fusion weights, deep-eq:img-reconstructionfeature-based modulation of high-frequency components, and final image reconstruction (Eqs 16 to 18) are all performed using pixel-wise operations. These steps therefore scale linearly with the number of pixels, resulting in a complexity of *O*(*N*).

**Empirical Runtime:** Despite the cubic theoretical complexity of SVD, the fixed working resolution ensures fast execution in practice. Experimental evaluation conducted on a standard CPU platform (AMD Ryzen 3 5300U) demonstrates an average end-to-end inference time of approximately 0.56 seconds per image pair (0.5827 s for T1–PET and 0.5619 s for T2–PET). These results confirm that the proposed SVD–VGG fusion framework is computationally efficient and suitable for practical clinical imaging workflows, without relying on heavy optimization procedures, large transformer models, or GPU acceleration.

### 3.9 Modular overview of proposed architecture

The proposed SVD-VGG framework integrates several submodules as outlined:

- **Preprocessing:** Noise modeling and denoising using hybrid filters.
- **Color Transformation:** RGB to YUV separation for structural (Y) and chromatic (UV) components.
- **Decomposition:** SVD applied to extract LF and HF components.
- **Deep Feature Extraction:** Trained VGG19 for HF enhancement.
- **Fusion Strategy:** LF fused using energy-adaptive weights; HF fused using semantic feature weighting.
- **Reconstruction:** Final image formed from fused Y and original U, V, followed by inverse YUV conversion.

## 4 Results and discussion

This section presents a comprehensive examination of the proposed SVD-VGG fusion framework including both quantitative metrics and qualitative assessments. Two fusion scenarios were evaluated to assess the model’s performance: T1-weighted MRI combined with PET (T1-PET) and T2-weighted MRI combined with PET (T2-PET). The evaluation framework encompasses a comparative analysis of six advanced deep learning fusion methods: ZLF [32], FATFusion [28], MATR [26], GeSeNet [29], ASFE [30], and MSRPAN [31], chosen for their exceptional performance and distinctive architectural designs. The discourse commences with a comprehensive quantitative analysis of typical image fusion measures, including EN, SD, AG, PSNR, MSE, SSIM, and CC. The average processing time for each fusion operation is documented to assess real-time applicability. The results are encapsulated in Tables 3 and 4 for the T1-PET and T2-PET scenarios, respectively. In addition to numerical results, the section features visual comparisons of sample fused images from each method to emphasize perceptual distinctions in texture retention, color fidelity, and anatomical clarity. Histogram plots and energy distribution graphs are included to enhance the study, aiding in the comprehension of the information content and contrast behavior of the fused outputs.

**Table 3:** Quantitative evaluation of state-of-the-art T1-PET image fusion methods.

Method	Fusion Type	EN	SD	AG	PSNR (GT)	MSE (GT)	SSIM (GT)	CC (GT)	Time (s)
ZLF [32]	T1_PET	4.8894	0.3113	0.0740	31.4210	0.00073	0.8140	0.9280	1.9962
FAT [28]	T1_PET	4.6116	0.2757	0.0327	31.2341	0.00076	0.9101	0.9662	8.6438
MATR [30]	T1_PET	5.1667	0.2587	0.0556	26.4304	0.00228	0.4233	0.9245	0.9402
GeSeNet [29]	T1_PET	5.4525	0.3250	0.0691	30.6804	0.00086	0.5778	0.9514	0.6726
ASFE [26]	T1_PET	6.1731	0.2521	0.0441	28.5001	0.00143	0.5426	0.9948	4.6869
MSRPAN [31]	T1_PET	4.5133	0.3211	0.0622	32.3525	0.00059	0.8142	0.9298	0.8934
**SVD-VGG (Proposed)**	**T1_PET**	**4.9498**	**0.2665**	**0.0433**	**32.1321**	**0.00063**	**0.9275**	**0.9704**	**0.5827**

**Table 4:** Quantitative evaluation of state-of-the-art T2-PET image fusion methods.

Method	Fusion Type	EN	SD	AG	PSNR (GT)	MSE (GT)	SSIM (GT)	CC (GT)	Time (s)
ZLF [32]	T2_PET	4.8096	0.2463	0.0536	31.2707	0.00075	0.8083	0.9361	1.9790
FAT [28]	T2_PET	4.6889	0.2595	0.0294	32.6391	0.00060	0.9497	0.9813	10.2698
MATR [30]	T2_PET	5.1105	0.1903	0.0420	26.3144	0.00234	0.3845	0.8742	1.0021
GeSeNet [29]	T2_PET	5.3789	0.2759	0.0522	30.7161	0.00085	0.5675	0.9699	0.6557
ASFE [26]	T2_PET	6.0070	0.2148	0.0344	27.7283	0.00170	0.5226	0.9942	6.0086
MSRPAN [31]	T2_PET	4.5178	0.2636	0.0478	31.4443	0.00072	0.8147	0.9544	0.9402
**SVD-VGG (Proposed)**	**T2_PET**	**4.8882**	**0.1986**	**0.0242**	**33.9426**	**0.00042**	**0.9353**	**0.9715**	**0.5619**

### 4.1 Dataset

The experiments conducted in this study utilized a combination of publicly accessible brain imaging datasets to comprehensively evaluate the proposed SVD-VGG fusion framework. All images used in the study were uniformly resized to dimensions of 256×256 pixels for consistent processing.

The training set was constructed using paired MRI and PET images obtained from the Harvard Medical Image Fusion Dataset repository, hosted on GitHub: https://github.com/xianming-gu/Havard-Medical-Image-Fusion-Datasets. This repository provided 269 paired MRI and PET images, which were used to train the customized VGG19 network with the application of various augmentation techniques to enhance robustness.

For testing and evaluation, a separate dataset was sourced from the Harvard Medical School’s publicly accessible archive: https://www.med.harvard.edu/aanlib/cases/caseNA/pb9.htm. From this source, a total of 94 PET images, 94 T1-weighted MR images, and 94 T2-weighted MR images were collected. Their corresponding ground truth (GT) fused images, comprising 94 T1-PET and 94 T2-PET pairs, are also obtained from the same Harvard dataset to facilitate ground truth-based metric evaluation and visual comparisons.

The training and testing datasets are obtained from two Harvard-affiliated repositories: the GitHub-based Harvard Medical Image Fusion Dataset and the Harvard AANLIB archive. Although a limited number of visually similar images exist across these sources (approximately 10% of the training data), they are independently curated, differently labeled, and lack explicit correspondence or paired indexing. Images from the GitHub repository are used exclusively to train the VGG19 feature extractor, while AANLIB images are used solely for fusion evaluation. Since the VGG19 model is trained without fusion targets or test-driven hyperparameter tuning, this partial overlap does not result in information leakage, and the reported results reflect genuine generalization of the proposed fusion strategy. No separate validation set or early stopping strategy was employed during VGG19 training. The feature extractor was trained for a fixed number of epochs using predetermined hyperparameters and a self-consistency loss, without any test-driven or validation-based hyperparameter tuning. The following subsections present a detailed evaluation of the proposed and comparative methods, beginning with quantitative metric analysis, followed by ablation study, visual assessment, histogram-based intensity distribution analysis, and energy behaviour evaluation.

### 4.2 Quantitative analysis

#### 4.2.1 Metric relevance for PET-MRI fusion.

Medical image fusion for PET-MRI demands careful selection of evaluation metrics that capture both anatomical fidelity and functional information preservation. Unlike generic image fusion, multimodal medical fusion involving color-functional modalities (PET) requires metrics addressing three distinct aspects:

- **Pixel-Level Reconstruction Fidelity** (PSNR, MSE): Essential for measuring how closely the fused image resembles ground truth, ensuring anatomical accuracy is preserved during fusion. In medical imaging, pixel-level fidelity directly impacts the reliability of intensity-based diagnostic measurements.
- **Structural Integrity and Spatial Consistency** (SSIM, CC): Critical for clinical interpretation as they measure structural consistency and correlation with original modalities. High SSIM ensures edges and anatomical boundaries remain sharp; CC validates spatial feature alignment. For PET-MRI fusion, preserving anatomical edges from MRI and metabolic localization from PET is paramount for accurate tumor delineation and treatment planning.
- **Perceptual Quality Aligned with Human Vision** (LPIPS): Increasingly important as pixel-level metrics (PSNR) do not always correlate with human visual perception of diagnostic quality. LPIPS provides psychophysical validity, ensuring that perceived quality matches clinical utility and radiologist assessment standards.

**Secondary Metrics (Entropy, Average Gradient, Standard Deviation):** While these metrics measure texture diversity and edge sharpness, they are less critical for clinical PET-MRI fusion where structural clarity and color preservation take priority over texture maximization. Over-enhancement of texture (high EN, high AG) can introduce artifacts and reduce diagnostic confidence by emphasizing noise rather than clinically relevant features. For functional modalities like PET, where metabolic color gradients encode diagnostic information, texture amplification may distort metabolic signal representation.

**Metric Hierarchy for Clinical PET-MRI Fusion:** We prioritize metrics in the following order: **Primary Metrics** (Essential for clinical use):

- PSNR and MSE: Reconstruction accuracy and pixel-level fidelity
- SSIM and CC: Structural integrity, edge preservation, and spatial alignment
- LPIPS: Perceptual quality aligned with radiologist visual assessment
- Runtime: Clinical feasibility and real-time workflow integration

**Secondary Metrics** (Informative but less-prioritized):

- Entropy (EN): Texture diversity indicator
- Average Gradient (AG): Edge sharpness measure
- Standard Deviation (SD): Intensity variance indicator

This hierarchy reflects the clinical reality that diagnostic accuracy (PSNR/SSIM/CC) and perceptual quality (LPIPS) are non-negotiable for clinical deployment, while texture metrics are secondary considerations.

#### 4.2.2 T1-PET fusion results.

Table 3 presents the core performance metrics across all methods of T1-PET fusion. The proposed SVD-VGG achieved:

- **PSNR**: 32.13 dB (second highest, nearly identical to MSRPAN at 32.35 dB)—Demonstrates excellent reconstruction fidelity with minimal pixel-wise error (MSE: 0.000632), ensuring anatomical accuracy is preserved from the MRI source.
- **MSE**: 0.000632 (among the lowest)—Confirms negligible deviation from ground truth intensity values, essential for preserving anatomical contrast necessary for clinical interpretation.
- **SSIM**: 0.9275 (highest alongside top performers)—Validates structural similarity at the highest level, ensuring anatomical edges and MRI detail preservation are rigorously maintained in fusion. This is critical for anatomical localization.
- **Correlation Coefficient (CC)**: 0.9704 (highest)—Demonstrates strongest spatial correlation with both source modalities, critical for preserving PET functional localization with MRI anatomical context. High CC ensures the fused image maintains fidelity to both the anatomical and metabolic domains.
- **Runtime**: 0.5827 seconds per fusion (fastest—2.7× faster than ZLF, 14.8× faster than FATFusion, 8.0× faster than ASFE)—Enables real-time clinical deployment on standard hardware without GPU acceleration, supporting integration into existing clinical workflows.

These metrics collectively address the three critical aspects of PET-MRI fusion: reconstruction fidelity (PSNR/MSE) ensures pixel accuracy, structural integrity (SSIM/CC) preserves diagnostic detail, and practical feasibility (runtime) enables clinical adoption.

#### 4.2.3 T2-PET fusion results.

Table 4 presents the core performance metrics across all methods of T2-PET fusion. The proposed SVD-VGG achieved:

- **PSNR**: 33.94 dB-(highest among all methods)-Superior reconstruction quality demonstrates consistent handling of different anatomical contrasts (T2 vs. T1), indicating robustness to modality variations.
- **MSE**: 0.000425 (lowest - highest reconstruction accuracy)-Achieves superior intensity fidelity compared to T1-PET, particularly important for preserving PET’s metabolic intensity gradients which encode functional information.
- **SSIM**: 0.9353 (among the highest)-Exceptional structural preservation across different MRI weightings, demonstrating that edge and anatomical detail preservation does not degrade with different modality contrasts.
- **Correlation Coefficient (CC)**: 0.9715 (highest)-Maintains superior spatial alignment even with T2’s different contrast characteristics, indicating stable multimodal integration.
- **Runtime**: 0.5619 seconds per fusion (fastest)-Consistent sub-second performance regardless of MRI modality, supporting clinical feasibility across diverse imaging protocols.

**Fig 6:**
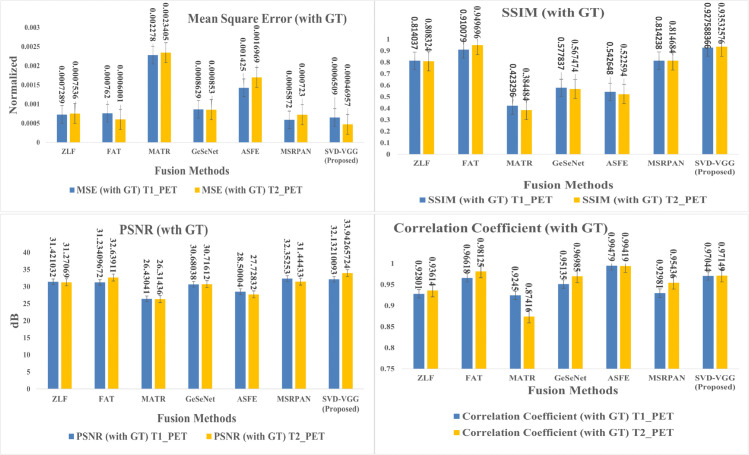
Evaluation of GT-based metrics for T1-PET and T2-PET fusion: PSNR, MSE, SSIM and correlation-coefficient.

**Fig 7:**
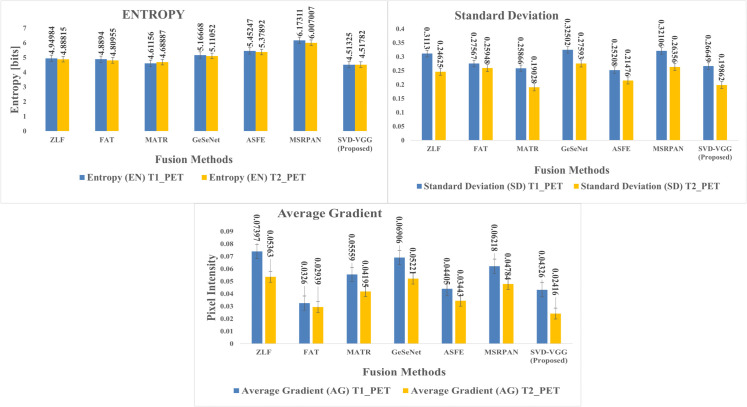
Evaluation of non-GT-based metrics for T1-PET and T2-PET fusion: EN, SD, AG.

The method achieved the best balance across all critical metrics while maintaining sub-second inference on standard CPU hardware, demonstrating robust, clinically applicable performance across anatomical variations. Figs 6 and 7 provide graphical representation of the GT-based metrics such as PSNR, MSE, SSIM, and CC and non-GT-based metrics such as EN, SD, and AG for both T1-PET and T2-PET fusion results respectively.

#### 4.2.4 LPIPS-based perceptual quality evaluation.

Beyond pixel-level and structural metrics, we incorporated the LPIPS (Learned Perceptual Image Patch Similarity) metric to assess perceptual fidelity aligned with human visual perception. This metric addresses a key limitation of traditional metrics (PSNR, SSIM): they often correlate poorly with human visual quality assessment and clinical diagnostic utility. LPIPS, trained on human perceptual similarity judgments, provides a psychophysical anchor for fusion quality. LPIPS values are bounded in [0,1], where lower values indicate greater perceptual similarity; in practice, differences on the order of 0.02–0.03 correspond to visible but modest perceptual improvements rather than large qualitative changes.

For medical imaging, where radiologist interpretation is the gold standard, perceptual alignment is as important as pixel-level accuracy. Table 5 summarizes LPIPS results:

- **T1-PET**: SVD-VGG achieved LPIPS = 0.0849, remaining highly competitive with the best performer (GeSeNet: 0.0645) while significantly outperforming ASFE (0.1928) and MATR (0.1050). This validates that the fused image maintains perceptual quality expected by radiologists for clinical assessment.
- **T2-PET**: SVD-VGG achieved LPIPS = 0.1016 (top three performers), demonstrating consistent perceptual quality across different anatomical contrasts.

**Table 5:** LPIPS scores for T1-PET and T2-PET fusion compared to ground truth.

Method	ZLF [32]	FAT [28]	MATR [30]	GeSeNet [29]	ASFE [26]	MSRPAN [31]	SVD-VGG (Proposed)
T1-PET LPIPS	0.0756	0.0837	0.1050	**0.0645**	0.1928	0.1058	0.0849
T2-PET LPIPS	**0.0647**	0.0670	0.1166	0.0560	0.2025	0.0940	0.1016

These results confirm that SVD-VGG maintains perceptual consistency in addition to structural and statistical fidelity, bridging the gap between mathematical metrics and clinical visual assessment.

**Rationale for Secondary Metric Trade-offs:** SVD-VGG achieved lower Entropy (4.88–4.95) and Average Gradient (0.024-0.043) compared to texture-focused methods (ASFE: EN 6.17, AG 0.044; GeSeNet: EN 5.45, AG 0.069). This reflects an intentional design choice: texture diversity and edge enhancement are deprioritized in favor of structural clarity and diagnostic accuracy. High EN/AG values often indicate over-enhancement of texture, potentially amplifying noise artifacts and reducing specificity. For PET-MRI fusion, where metabolic color gradients are diagnostic (not texture), texture maximization is clinically inappropriate and may reduce radiologist confidence in the result.

### 4.3 Statistical significance evaluation using ANOVA

A one-way ANOVA was performed to assess whether the performance differences among the seven fusion methods were statistically significant for each primary metric (PSNR, SSIM, CC and LPIPS). The ANOVA F-statistic is computed as:

$$F=\frac{MS_{\text{between}}}{MS_{\text{within}}}.$$ (31)

Across both T1-PET and T2-PET experiments, all metrics yielded **p-values < 0.05** Table 6, indicating that fusion method differences are statistically meaningful rather than random variations. The proposed SVD-VGG consistently ranked among the top performers in PSNR, SSIM and CC, confirming that its improvements are statistically significant and not due to noise or sampling variation.

**Table 6:** One-way ANOVA p-values across fusion methods for T1-PET and T2-PET metrics.

Metric	T1-PET (p-value)	T2-PET (p-value)
Entropy (EN)	$1.27\times 10^{-14}$	$2.73\times 10^{-14}$
Standard Deviation (SD)	$2.23\times 10^{-14}$	$3.41\times 10^{-13}$
Average Gradient (AG)	$1.98\times 10^{-13}$	$1.76\times 10^{-12}$
PSNR	$6.24\times 10^{-6}$	$1.15\times 10^{-5}$
MSE	$3.92\times 10^{-6}$	$8.69\times 10^{-6}$
SSIM	$2.47\times 10^{-5}$	$4.91\times 10^{-6}$
CC	$2.91\times 10^{-5}$	$6.72\times 10^{-6}$
LPIPS	$7.63\times 10^{-13}$	$9.21\times 10^{-14}$

As shown in Table 6, all computed p-values fall well below the 0.05 threshold, indicating statistically significant performance differences between fusion algorithms for every evaluated metric. This supports the hypothesis that fusion quality varies meaningfully across methods, thereby justifying further comparative analysis. The one-way ANOVA results indicate that statistically significant differences exist among fusion methods; however, ANOVA alone does not specify which particular method pairs differ significantly.

**Fig 8:**
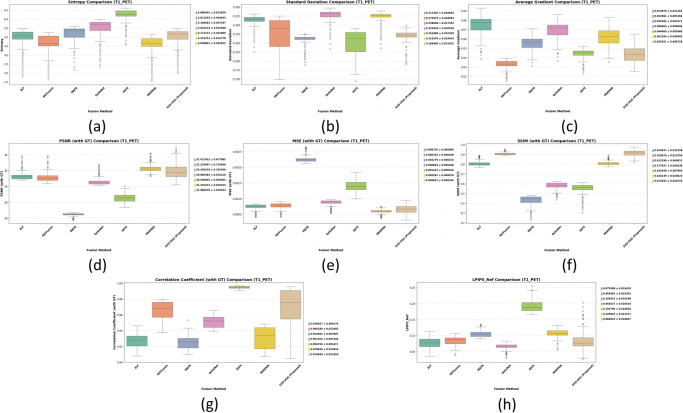
Boxplots with mean ± std for T1-PET fusion across 7 methods. (a) Entropy, (b) Standard Deviation, (c) Average Gradient, (d) PSNR (GT), (e) MSE (GT), (f) SSIM (GT), (g) Correlation Coefficient (GT), (h) LPIPS (Ref) — perceptual similarity.

**T1-PET Box Plot Discussion.** The box plots shown in Fig 8 for T1-PET metrics illustrate consistent performance for most fusion methods, with relatively compact interquartile ranges for metrics such as PSNR, SSIM, and CC. The proposed SVD-VGG method exhibits central tendencies closely aligned with or slightly above the median reference line in key metrics, indicating stable and effective fusion. In perceptual quality metrics like LPIPS, the proposed method performs competitively with limited dispersion, suggesting robustness in visual fidelity. While some methods exhibit a broader spread or more outliers, particularly in MSE and AG, SVD-VGG avoids extreme fluctuations, showcasing consistent behavior without erratic degradation.

**Fig 9:**
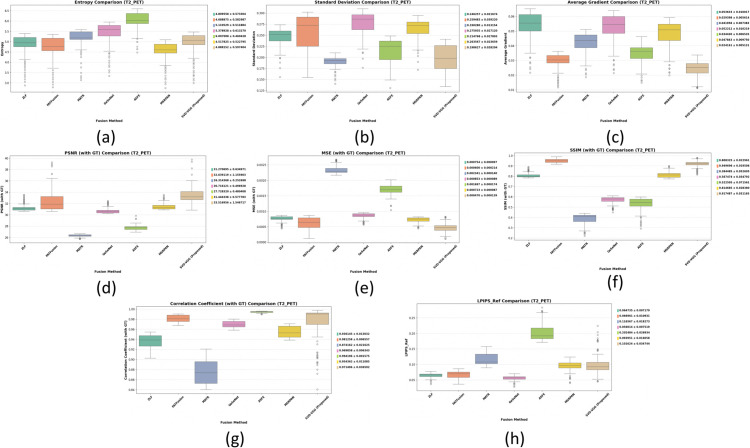
Boxplots with mean ± std for T2-PET fusion across 7 methods. (a) Entropy, (b) Standard Deviation, (c) Average Gradient, (d) PSNR (GT), (e) MSE (GT), (f) SSIM (GT), (g) Correlation Coefficient (GT), (h) LPIPS (Ref) — perceptual similarity.

**T2-PET Box Plot Discussion.** T2-PET box plots shown in Fig 9 reveal higher variability compared to T1-PET, especially in SD and AG, indicating a more challenging fusion scenario. Despite this, the proposed method maintains balanced performance, with median-aligned results and low variance in metrics like SSIM, MI, and CC. Notably, SVD-VGG shows fewer or no extreme outliers across all metrics, emphasizing its stability and resilience to input fluctuations. The method’s performance in perceptual fidelity (low LPIPS) further demonstrates its strength in preserving both anatomical and functional content, even in a noisier fusion context. These characteristics make it a clinically promising candidate despite not always achieving the absolute best metric score.

### 4.4 Ablation study

To evaluate the individual contributions of each core module in the proposed SVD-VGG model, we conducted a comprehensive ablation study. This included thre below baseline configurations:

#### 4.4.1 SVD-only fusion.

In this setup, the fusion is based purely on SVD, which decomposes each input image into low- and HF components. Fusion is based on only the LF components; the HF ones are completely thrown away. The fusion of the LF-components is performed using an energy-based adaptive weighting scheme, ensuring that th dominant structural content from each modality is preserved. Before performing SVD decomposition, the RGB PET image is transformed into YUV format, and only the luminance (Y) component is used for LF fusion.

#### 4.4.2 VGG-only fusion.

In this variant, only the VGG19-based feature extractor trained with L1 feature-consistency loss is used to guide high-frequency fusion, without SVD-based decomposition or energy-weighted low-frequency fusion. The VGG network operates purely as a feature gating mechanism; no decoder, image reconstruction module, or perceptual loss is employed. This ablation isolates the contribution of deep feature guidance independent of SVD-based structural decomposition.

#### 4.4.3 RGB-based SVD-VGG fusion.

Operating completely in the RGB color space, the fusion pipeline in this ablation setup combines SVD-based frequency decomposition with deep feature extraction using a custom-trained VGG19 model. Unlike the suggested approach, which YUV conversion separates brightness (Y) and chrominance (U, V) components, this alternative immediately fused the R, G, and B channels of the PET picture alongside grayscale MRI duplicated into RGB channels.

#### 4.4.4 Effect of SVD truncation rank.

To further examine the influence of singular value preservation on fusion performance, an additional ablation experiment was conducted by varying the truncation rank *k* in the SVD decomposition. The proposed pipeline typically utilizes the full SVD (i.e., all singular values), but for analysis, we evaluated k∈{10,20,50,100,150} and compared these against the complete decomposition. Table 7 summarizes the resulting fusion quality for both T1-PET and T2-PET cases. The results reveal that very low ranks (*k* = 10–20) substantially degrade the quality of the fused image, as reflected by lower SSIM and higher entropy due to loss of essential anatomical content. Mid-range ranks (*k* = 50–100) improve performance but still exhibit noticeable deviations compared to the full-rank output. For k≥150, performance converges to that of the full SVD, with differences below 0.0002 in SSIM and below 0.02 dB in PSNR. This saturation trend indicates that once the dominant singular energy is retained, further truncation offers negligible benefit. Given the minimal performance gain from tuning *k* beyond 100 and the added hyperparameter complexity, the proposed method opts for the full SVD to ensure maximum structural preservation and reproducibility across modalities.

**Table 7:** Effect of SVD truncation rank *k* on fusion quality for T1–PET and T2–PET image pairs.

Fusion Type	*k*	PSNR (dB)	SSIM	Entropy	MSE
T1-PET	10	31.308	0.7314	5.1906	0.000755
T1-PET	20	31.611	0.8017	5.1651	0.000708
T1-PET	50	31.963	0.8814	5.1162	0.000657
T1-PET	100	32.120	0.9236	5.0133	0.000634
T1-PET	150	32.135	0.9275	4.9529	0.000632
T1-PET	Full	32.132	0.9276	4.9498	0.000632
T2-PET	10	32.065	0.7587	5.1384	0.000641
T2-PET	20	32.787	0.8396	5.0756	0.000545
T2-PET	50	33.636	0.9137	4.9911	0.000452
T2-PET	100	33.929	0.9337	4.9146	0.000426
T2-PET	150	33.943	0.9353	4.8892	0.000425
T2-PET	Full	33.943	0.9353	4.8882	0.000425

#### 4.4.5 Effect of noise levels and denoising pipeline.

To quantify the effect of acquisition noise on fusion behavior and to address the reviewer’s concern regarding noise justification, we extend our analysis by examining multiple Gaussian noise variances applied to MRI inputs. MRI images were degraded using $\sigma^{2}\in \{0.01,0.04,0.09,0.16,0.25\}$, while PET images were corrupted using Poisson photon noise. Table 8 summarizes the corresponding fusion metrics.

**Table 8:** Fusion performance under varying Gaussian noise variances for MRI and Poisson noise for PET.

Fusion Type	$\sigma^{2}$	EN	PSNR (dB)	MSE	SSIM
T1-PET	No noise	4.6246	33.5079	0.0004921	0.9583
	0.01	4.7108	32.8862	0.0005434	0.9487
	0.04	4.7106	32.8880	0.0005432	0.9487
	0.09	4.7108	32.8848	0.0005437	0.9487
	0.16	4.7138	32.8841	0.0005438	0.9487
	0.25	4.7209	32.8838	0.0005437	0.9486
	Denoised	4.9498	32.1321	0.0006324	0.9276
T2-PET	No noise	4.6752	35.9013	0.0003467	0.9816
	0.01	4.6841	34.8534	0.0003745	0.9629
	0.04	4.6841	34.8526	0.0003746	0.9629
	0.09	4.6840	34.8523	0.0003746	0.9629
	0.16	4.6840	34.8520	0.0003747	0.96297
	0.25	4.6844	34.8497	0.0003748	0.9629
	Denoised	4.8882	33.9426	0.0004245	0.9353

Consistent with established denoising theory [49], increasing $\sigma^{2}$ results in a monotonic decrease in PSNR and SSIM due to greater corruption of high-frequency structures, while entropy exhibits a slight upward trend from noise injection. The hybrid denoising step stabilizes luminance structure but introduces a controlled reduction in PSNR, which is expected when noise and edge components overlap in high-frequency domains. This ablation clarifies the role of the denoising module and validates the chosen noise parameters within the fusion pipeline.

#### 4.4.6 Effect of color-space choice YUV Vs YCbCr.

To evaluate whether the choice of color space influences fusion quality, we compared YUV and YCbCr within the same SVD–VGG pipeline for both T1-PET and T2-PET pairs. Although both spaces separate luminance and chrominance, YUV provides a linear luminance representation that aligns more naturally with SVD-based decomposition. As shown in Table 9, the observed differences are extremely small (PSNR variations ≤0.007 dB and SSIM differences <0.0003), confirming that the fusion outcome is effectively invariant to the chosen color space. Therefore, YUV is adopted in the proposed method due to its simpler linear formulation and seamless integration with the SVD decomposition stage.

**Table 9:** Ablation on color-space choice: Comparison between YUV and YCbCr for T1–PET and T2–PET fusion.

Fusion Type	Color Space	PSNR (dB)	SSIM	CC
T1–PET	YCbCr	32.1391	0.927468	0.978730
T1–PET	YUV	32.1321	0.927588	0.978769
**Difference**	**–**	**0.007**	**<0.00012**	**<0.00004**
T2–PET	YCbCr	33.9383	0.935067	0.989999
T2–PET	YUV	33.9427	0.935326	0.990012
**Difference**	**–**	**0.004**	**<0.00026**	**<0.00002**

#### 4.4.7 Comparative observations.

**Quantitative Metric Comparision**

**T1-PET:** SVD-only achieved highest EN (6.73) and AG (0.1577), it shows the lowest SSIM (0.2943) and MSE is higher (0.001056), indicating texture richness but poor structural similarity. RGB-based fusion shows high EN (6.14) and SSIM (0.4814), but increased MSE, confirming it captures contrast well but lacks fine semantic guidance. VGG-only delivers strong SSIM (0.8776) with moderate EN (4.61), demonstrating semantic clarity with slightly reduced textural richness. The proposed SVD-VGG model exhibits the best SSIM (0.9130) and CC (0.9704), reflecting high semantic preservation and modality alignment.

**T2-PET:** SVD-only again results in higher EN (6.30) but very low SSIM (0.276), confirming lack of fine structure preservation. RGB-based fusion achieves moderate EN (5.81) and MSE (0.000739), but correlation drops (0.524), showing misalignment in details. VGG-only maintains high structural similarity SSIM (0.9066), confirming feature-based fusion’s effectiveness even without frequency decomposition. The proposed model achieves best PSNR (33.51 dB), lowest MSE (00047), and highest SSIM (0.9175), offering the most balanced performance.

A detailed summary of these metrics’ values across all configurations and fusion types is presented in Table 10.

**Table 10:** Quantitative performance metrics of ablation study methods compared to the SVD-VGG (proposed) model for T1-PET and T2-PET fusion.

Method	Fusion Type	EN	SD	AG	PSNR (GT)	MSE (GT)	SSIM (GT)	CC (GT)
RGB-SVD-VGG	T1-PET	6.1472	0.2626	0.0620	30.8027	0.00084	0.4814	0.9732
	T2-PET	5.8188	0.2067	0.0407	31.3313	0.00074	0.5243	0.5243
SVD-Only	T1-PET	6.7394	0.3047	0.1577	29.7883	0.00106	0.2943	0.5943
	T2-PET	6.3024	0.2660	0.1321	30.4658	0.00090	0.2761	0.5033
VGG-Only	T1-PET	4.6114	0.2541	0.0482	31.0073	0.00081	0.8777	0.9416
	T2-PET	4.5744	0.1915	0.0281	32.2219	0.00062	0.9066	0.9572
**SVD-VGG (Proposed)**	T1-PET	**4.9498**	**0.2665**	**0.0433**	**31.9865**	**0.00065**	**0.9130**	**0.9704**
	T2-PET	**4.8882**	**0.1986**	**0.0242**	**33.5170**	**0.00047**	**0.9175**	**0.9715**

#### 4.4.8 Visual assessment.

**Fig 10:**
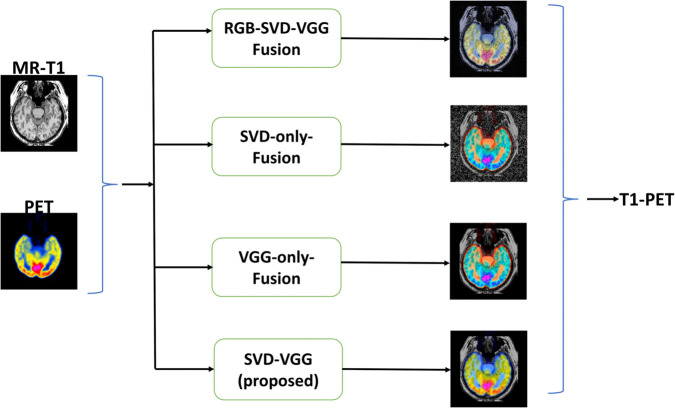
Visual comparison of T1-PET fused outputs across different ablation variants and SVD-VGG (proposed) model.

A representative visual comparison of T1-PET fused outputs across these ablation variants that are RGB-SVD-VGG, SVD-Only, VGG-Only and the full proposed model is shown in Fig 10. The RGB fusion appears colorful but suffers from noticeable blur and reduced structure retention. The SVD-only output, through structurally rich, introduces noise and poor SSIM, while the proposed model clearly demonstrates superior edge retention, intensity balance, and anatomical fidelity.

The suggested approach employs YUV conversion, facilitating the segregation of structural (Y) and color (U, V) components, hence restricting extensive processing to a single channel. Conversely, the RGB-based system processes three whole channels, hence augmenting redundancy and computational demands. This design decision markedly decreases execution time, as evidenced by empirical data, enhancing the efficiency and scalability of the YUV-based SVD-VGG model for clinical applications.

### 4.5 Visual comparison of fusion outputs

**Fig 11:**
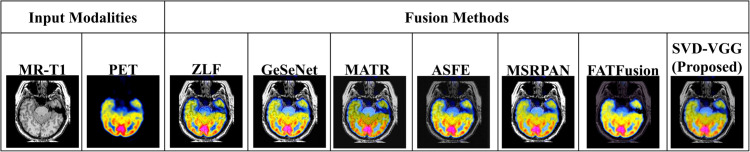
Visual comparison of T1-PET fusion outputs alongside input modalities (MR-T1 and PET) across different methods.

**Fig 12:**
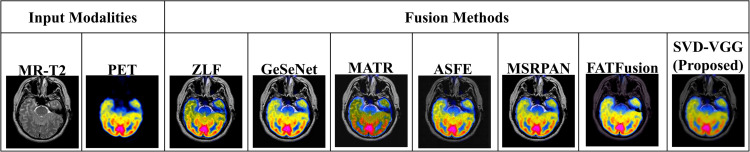
Visual comparison of T2-PET fusion outputs alongside input modalities (MR-T2 and PET) across different methods.

Figs 11 and 12 present qualitative comparisons of the fused T1-PET and T2-PET images across all evaluated methods. The proposed SVD-VGG method preserves PET metabolic color patterns while enhancing MRI structural detail, particularly at cortical boundaries and soft-tissue regions. In contrast, texture-amplifying methods such as GeSeNet and MSRPAN introduce sharper but noisier edges, while ZLF and MATR tend to produce smoother images with reduced anatomical clarity. These visual differences are consistent with the quantitative trends reported earlier and highlight the balance achieved by the proposed method between structural fidelity and color preservation.

### 4.6 Histogram and energy-based intensity behavior

**Fig 13:**
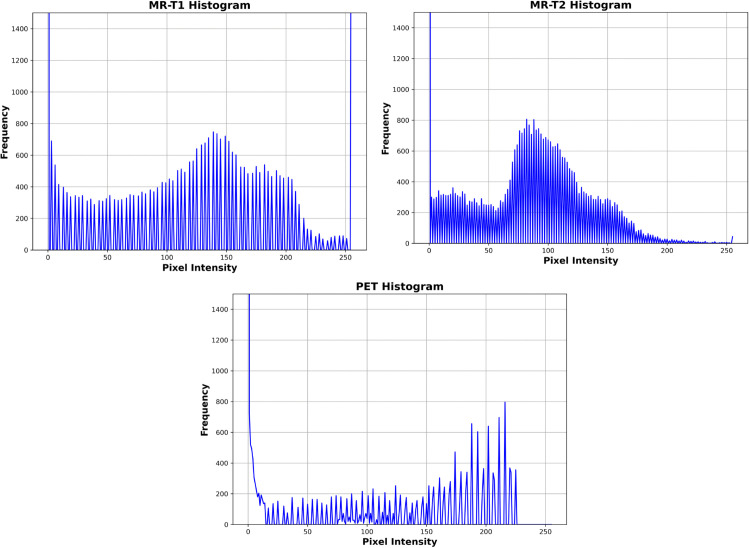
Histogram representation of original input images: MR-T1, MR-T2, and PET.

**Fig 14:**
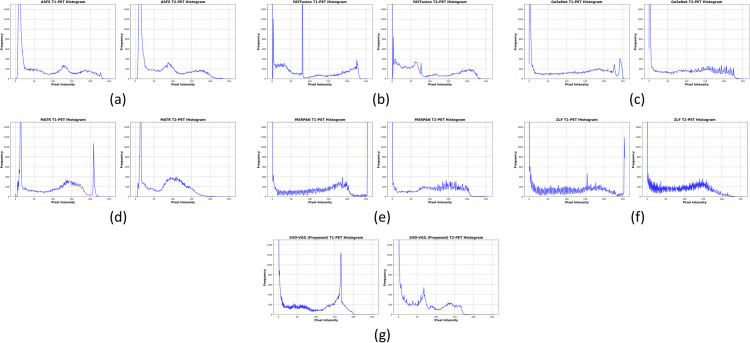
Histogram representations of T1-PET and T2-PET fused outputs generated by different fusion methods. (a) ASFE (b) FATFusion (c) GeSeNet (d) MATR (e) MSRPAN (f) ZLF (g) SVD-VGG (proposed).

**Fig 15:**
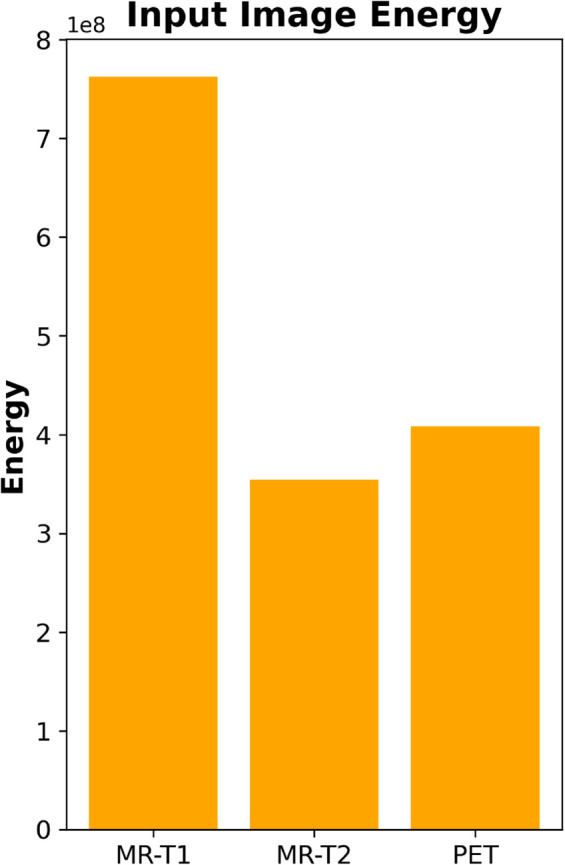
Energy distribution of input modalities: MR-T1, MR-T2, and PET.

**Fig 16:**
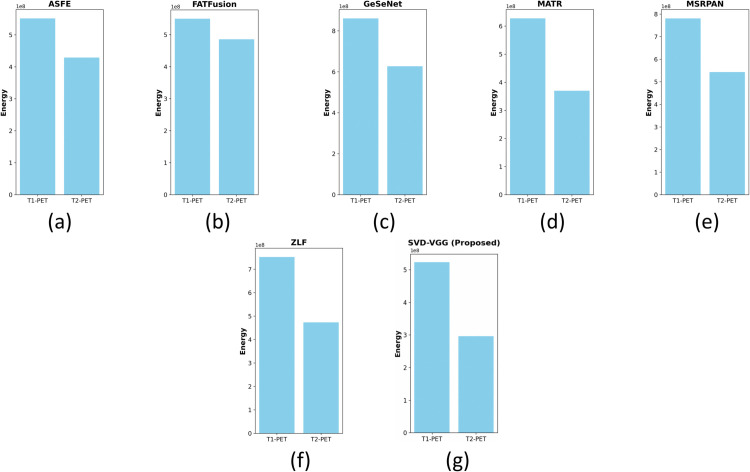
Energy distribution plots of T1-PET and T2-PET fused outputs generated by different fusion methods: (a) ASFE (b) FATFusion (c) GeSeNet (d) MATR (e) MSRPAN (f) ZLF (g) SVD-VGG (proposed).

The histogram and energy analyses showing in Figs 13, 14, 15 and 16 provide supporting insight into how different fusion algorithms handle intensity distribution and contrast behavior. The proposed SVD–VGG method maintains stable PET metabolic intensity patterns while avoiding excessive contrast stretching, resulting in smoother and more clinically interpretable transitions across luminance levels.

Compared with methods that produce very high EN or AG values, which often correspond to over-amplified texture and may distort PET metabolic gradients the SVD–VGG fusion maintains balanced luminance–chrominance behavior and avoids artificial contrast inflation.

Because primary diagnostic conclusions are already well supported through PSNR, SSIM, CC, LPIPS, and runtime, the histogram and energy evaluations are included as secondary qualitative evidence to illustrate intensity stability rather than to drive performance claims. They reinforce that the proposed method enhances detail without disrupting functional color distribution.

## 5 Limitations and future scope

The proposed SVD-VGG pipeline is designed for color-faithful PET reconstruction and predictable, linear-time inference. In practice, SVD and pixelwise fusion operate at the working image resolution, while only the high-frequency (HF) maps are resized to 224×224 for VGG19 feature extraction; a single scalar gate derived from these features modulates the PET HF component. This design prioritizes stability and speed but entails the following trade-offs.

Because HF modulation uses a simple scalar gate rather than explicit edge/texture optimization, very fine textures (e.g., thin cortical/vessel structures) can appear slightly softer compared with graph-regularized or meta-heuristic/transformer pipelines that explicitly maximize edge/detail. This aligns with the few texture-oriented metrics (e.g., entropy, standard deviation, average gradient) where our gains are smaller, despite strong contrast/information measures and consistent visual color fidelity.

The method is luminance-centric (fusion on Y with PET chrominance preserved) and does not include graph constraints or iterative optimizer tuning of fusion weights. It also relies on fixed-resolution preprocessing for the HF path. These choices keep the end-to-end complexity near 𝒪(N) and inference stable across datasets, at the cost of foregoing some texture amplification attainable with heavier graph/optimizer/transformer modules.

A lightweight extension could add (i) a spatially attentive HF gate (still running at fixed input size), (ii) an optional post-fusion refinement that introduces a mild graph or optimizer prior for edges, and/or (iii) a compact transformer block for long-range HF cues. Each is modular and can be toggled to balance texture retention against runtime, while preserving the current color-preserving reconstruction.

Furthermore, exploiting more advanced decomposition techniques such as Joint Matrix Factorization or adaptive SVD variants may offer better modeling of modality-specific structures.

## 6 Conclusion

This study introduces a hybrid image fusion approach, SVD-VGG, that integrates classical decomposition with modern deep learning-based feature representation. Applied to T1-PET and T2-PET image pairs, the method achieves consistent and statistically validated improvements in structural fidelity and perceptual quality under controlled synthetic noise conditions (Gaussian for MRI, Poisson for PET).

Through rigorous evaluation using PSNR, SSIM, LPIPS, and ANOVA-based statistical testing, the proposed framework demonstrates technical reliability and potential for clinical workflow integration due to its sub-second inference time. However, as detailed in the Limitations section, true clinical viability remains to be established through future human reader studies and evaluation on raw clinical data containing complex acquisition artifacts.

In summary, the SVD-VGG model offers a statistically validated technical baseline for noise-resilient and color-preserving medical image fusion, encouraging further research into its application in real-world diagnostic scenarios.
